# Resveratrol reduces drug resistance of SCLC cells by suppressing the inflammatory microenvironment and the STAT3/VEGF pathway

**DOI:** 10.1002/2211-5463.13230

**Published:** 2021-07-16

**Authors:** Cong Hou, Lijun Lu, Zhanye Liu, Yingjie Lian, Jianguang Xiao

**Affiliations:** ^1^ Department of Cardiothoracic Surgery Zoucheng People's Hospital Jining City China; ^2^ Department of Thoracic Surgery Caoxian People's Hospital Heze City China; ^3^ Department of Thoracic Surgery Laizhou People's Hospital Laizhou City China

**Keywords:** Adriamycin, H69AR cells, IL‐23, resveratrol, small cell lung cancer, STAT3/VEGF signaling pathway

## Abstract

DNA‐damaging agents, such as doxorubicin (Adriamycin), are widely used for the treatment of small cell lung cancer (SCLC). However, drug resistance is one of the major challenges for treatment of SCLC. Herein, we investigated the mechanisms underlying drug resistance in SCLC cells and the effects of resveratrol (Res) on drug resistance. We report that Adriamycin treatment of H69AR (multidrug resistance phenotype) cells resulted in a lower rate of growth inhibition, up‐regulation of MRP1 and P‐glycoprotein (P‐gp), and higher P‐gp activity as compared with susceptible H69 cells treated with Adriamycin. Moreover, the signal transducer and activator of transcription 3/vascular endothelial growth factor (STAT3/VEGF) pathway was overactivated in H69AR cells, especially after interleukin‐23 treatment. The inflammatory microenvironment promoted the drug resistance of H69AR cells by activating the STAT3/VEGF pathway. The addition of Res suppressed the expression levels of inflammatory mediators, inhibited the STAT3/VEGF pathway, impeded P‐gp activity, and decreased the drug resistance of H69AR cells. H69AR cells exhibited Adriamycin resistance through activation of the STAT3/VEGF pathway, and Res ameliorated the inflammatory microenvironment to suppress the STAT3/VEGF pathway to reduce drug resistance. Our results suggest that Res may have therapeutic potential for SCLC treatment.

## Graphical abstract

Resveratrol inhibits the expression and secretion of proinflammatory proteins to improve the inflammatory microenvironment via reducing the activation of nuclear factor‐κB. It also blocks interleukin‐23‐induced excessive activation of the signal transducer and activator of transcription 3 (STAT3)/vascular endothelial growth factor signaling pathway by inhibiting STAT3 phosphorylation, thereby reducing multidrug resistance 1 transcription to down‐regulate the expression levels of MRP1 and P‐glycoprotein; in turn, P‐glycoprotein plays a key role in drug efflux leading to Adriamycin resistance.

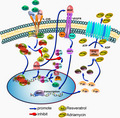

**Abbreviations**
IL‐23: interleukin‐23
MDR1: multidrug resistance 1
P‐gp: P‐glycoprotein
Res: resveratrol
SCLC: small cell lung cancer
SD: standard deviation
si‐NC: negative control siRNA
siRNA: small interfering RNA
si‐STAT3: specific siRNA targeting STAT3
STAT3: signal transducer and activator of transcription 3
VEGF: vascular endothelial growth factor

Lung cancer with the leading cancer‐related mortality rate worldwide can be divided into small cell lung cancer (SCLC) and non‐SCLC [1]. SCLC is an aggressive disease of neuroendocrine origin that accounts for 15–20% of all lung carcinomas [2, 3, 4, 5]. SCLC is considered different from the other lung cancer types for its early distant metastasis, aggressive growth and highest malignancy [2, 5, 6]. Clinically, it has been proved that the poor prognosis of SCLC is probably due to the rapid evolution from chemosensitivity to drug resistance [7]. The widely used drugs for the treatment of SCLC are DNA‐damaging agents [8], such as doxorubicin (Adriamycin), which show a potent effect on preventing cell division and enhancing cell death [9]. However, the cells often acquire drug resistance, but the molecular mechanisms of drug resistance are not fully elucidated in SCLC.

Inflammation can enhance cell proliferation and the production of survival signals to promote the development of tumors [10]. Not only does inflammation show a procancer effect but also it induces angiogenesis by influencing immune regulation [10]. Interleukin‐23 (IL‐23), which is overexpressed in lung cancer, is significantly associated between IL‐23 and the recurrence and prognosis of SCLC [11]. It has been reported that IL‐23 can mediate inflammatory processes, providing a tumor microenvironment by activating the signal transducer and activator of transcription 3 (STAT3) signaling pathway in tumors [12, 13].

In several types of cancer, including lung cancer, the malignant transformation is implicated with the STAT3 signaling pathway by constitutively activating the transcription factor signal transducer and STAT3 [14]. The STAT3 signaling pathway is activated by STAT phosphorylation, which increases the expression levels of target genes, such as vascular endothelial growth factor (VEGF), in all tumor growth processes. In addition, recombinant human VEGF increases the expression of MRP1 or enhances the activity of MRP1 in K562 and BGC‐823 cell lines [15]. Multidrug resistance 1 (*MDR1*), a multidrug resistance gene in tumor cells, produces P‐glycoprotein (P‐gp) that alters intracellular drug distribution. Fabbro *et al*. [16] have proved that VEGFR‐2 and PKC412 inhibitors reverse the P‐gp‐mediated MDR role in cancer cells by blocking the VEGF cellular signaling pathway. Hence we conjecture that overactivation of the STAT3/VEGF signaling pathway was associated with drug resistance of SCLC.

The high expression level of *MDR1* is ubiquitous in various cancers or diseases with drug resistance. In pediatric soft tissue sarcoma, the *MDR1* gene exerts a predominant role in innate drug resistance [17]. Meanwhile, the high expression level of *MDR1* is partially responsible for drug resistance of Burkitt lymphoma cells [18]. Pop *et al*. [19] have revealed that overexpression of *MDR1*/P‐gp is inhibited to overcome the vincristine‐resistant effects in B lymphocytes, thereby restoring the sensitivity of anticancer and antimicrobial drugs. Besides, cisplatin resistance in bladder cancer cells is reversed via reducing *MDR1* expression [20]. Another example is that in the Adriamycin‐resistant human hepatic carcinoma mouse model, the antitumor efficacy of Adriamycin is reversed by *MDR1* small interfering RNA (siRNA) [21].

Resveratrol (Res) has many therapeutic functions, including antiapoptosis, antioxidation, antitumor and anti‐inflammation roles [22, 23]. Recent evidence has shown that Res plays an antitumor effect in SCLC [24]. A study has shown that Res increases the chemotherapy sensitivity of cholangiocarcinoma [25]. Besides, the negative regulation of the STAT3 pathway can overcome the drug resistance in multiple myeloma cells [26] and prostate cancer cells [27]. However, whether Res can overcome drug resistance and increase drug sensitivity in SCLC cells under an inflammatory microenvironment has not been reported. This study aims to investigate the mechanism of the inflammatory microenvironment on drug resistance in SCLC cells and the effects of Res.

## Materials and methods

### Cell culture

Human SCLC cell lines H69 and H69AR (the MDR phenotype) were obtained from the American Type Culture Collection (Manassas, VA, USA). Then the cells were maintained in RPMI 1640 containing 10% FBS (Thermo Fisher, Waltham, MA, USA) and 1% penicillin–streptomycin under 5% CO_2_ at 37 °C.

### MTT assay

To measure the viability of H69 and H69AR cells, we used the MTT Kit (Sigma, St. Louis, MO, USA). When cells were at the logarithmic growth phase, they were plated (1.5 × 10^4^ cells per well) and treated with Adriamycin (Selleck Chemicals, Houston, TX, USA) alone at different concentrations or Adriamycin combined with Res (Alexis Biochemicals, San Diego, CA, USA) and/or IL‐23, respectively. After treatment for 24 and 48 h, MTT was added and maintained for 4 h. Then we applied 200 μL DMSO to replace the culture medium, and a microplate reader (Bio‐Rad, Shanghai, China) was used to read the absorbance at 492 nm (*A*_492 nm_). The cell growth inhibition rate was calculated according to the formula: (1 − absorbance of reagent‐treated cells/absorbance of untreated control cells) × 100%.

### STAT3 knockdown

The specific siRNA targeting STAT3 (si‐STAT3) and the negative control siRNA (si‐NC) were obtained from GenePharma (Shanghai, China). The H69AR cells were transfected with si‐STAT3 using Lipofectamine 3000 (Invitrogen, Carlsbad, CA, USA). Then the cells were labeled as H69AR group, si‐NC group and si‐STAT3 group for subsequent tests. Forty‐eight hours after transfection, cells were collected for determination of siRNA transfection efficiency.

### Quantitative real‐time PCR

The relative expression level of *MDR1* was measured by quantitative real‐time PCR. The TRIzol Reagent Kit (Takara, Otsu, Japan) was applied to isolate total RNA, and the miRNA First Strand cDNA synthesis kit (Sangon Biotech, Shanghai, China) was applied to transcribe RNA into cDNA. Quantitative real‐time PCR was performed with SYBR® Prime Script™ RT‐PCR Kit (Invitrogen). The primer sequences were as follows: *MDR1* forward: 5′‐CCCATCATTGCAATAGCAGG‐3′, reverse: 5′‐ TGTTCAAACTTCTGCTCCTGA‐3′; *GAPDH* forward: 5ʹ‐GTCTCCTCTGACTTCAACAGCG‐3ʹ, reverse: 5ʹ‐ACCACCCTGTTGCTGTAGCCAA‐3ʹ. The Mx3000P real‐time PCR system (Thermo Fisher) was used. The PCR conditions were described as follows: 95 °C for 15 min and then 40 cycles of 94 °C for 15 s, 60 °C for 1 min and 72 °C for 1 min. All procedures were conducted in triplicate. The 2^−ΔΔCt^ method was used to calculate the relative *MDR1* expression.

### Western blot

The protein expression levels were measured by western blot. In brief, total protein was obtained using the specific protein extraction kit (BestBio Institute of Biotechnology, Wuhan, China). The amounts of total protein were quantified by the BCA assay (Keygen Institute of Biotechnology, Nanjing, China). Protein was resolved by 6–15% SDS/PAGE and transferred onto poly(vinylidene difluoride) membranes (EMD Millipore, Billerica, MA, USA), then blocked with 5% nonfat milk. Primary antibodies, including P‐gp (1 : 1000; ab170904; Abcam, Cambridge, UK), STAT3 (1 : 1000; ab76315; Abcam), IL‐8 (1 : 1000; ab18672; Abcam), IL‐23 (1 : 1000; ab45420; Abcam), VEGF (1 : 1000; ab46154; Abcam), p‐STAT3 (1 : 1500; 9145; Cell Signaling Technologies, Danvers, MA, USA), MRP1 (1 : 1000; 72202; Cell Signaling Technologies), IL‐1β (1 : 1000; 12703; Cell Signaling Technologies) and p‐NF‐κB (1 : 800; sc‐136548; Santa Cruz Technology, Santa Cruz, CA, USA), were added and incubated overnight at 4 °C. The anti‐IgG secondary antibodies (ab205718, ab190475; Abcam) were subsequently applied to the membranes and incubated for 2 h. Immunoreactive signals were revealed by the enhanced chemiluminescence detection system (GE Healthcare, Muenchen, Germany). imagej software (National Institute of Health, Bethesda, MD, USA) was applied to analyze protein expressions.

### Flow cytometry

The apoptosis of H69 and H69AR cells was measured by flow cytometry. In brief, the cells were collected after treatment with Adriamycin, or its combination with Res and/or IL‐23, and incubated with ECD (5 μL, phycoerythrin‐Texas Red conjugate (the energy coupled dye)) and Annexin V–FITC (5 μL) solution (BD Biosciences, Franklin Lakes, NJ, USA) for 15 min. The cell apoptosis rates were analyzed under a flow cytometer (BD Biosciences).

### Rhodamine‐123 accumulation assay

The accumulation of rhodamine‐123, which reflected P‐gp activity, was detected in H69 and H69AR cells. In brief, after treatment, the H69 and H69AR cells were treated with rhodamine‐123 (5 μm; Sigma Chemical Company) and incubated at 37 °C for 2 h. Then cold PBS and 1% Triton X‐100 were used to wash H69 and H69AR cells. The accumulation of rhodamine‐123 was measured using a confocal laser scanning microscope (Olympus, Tokyo, Japan). imagej software was applied to analyze the earlier data.

### Statistical analysis

All the experiments were performed in triplicate and repeated at least three times. The data were presented as mean ± standard deviation (SD). Student’s unpaired *t*‐test and one‐way ANOVA were used for statistical analysis. *P* < 0.05 was considered statistically significant.

## Results

### The Adriamycin resistance of H69AR cells

**Fig. 1:**
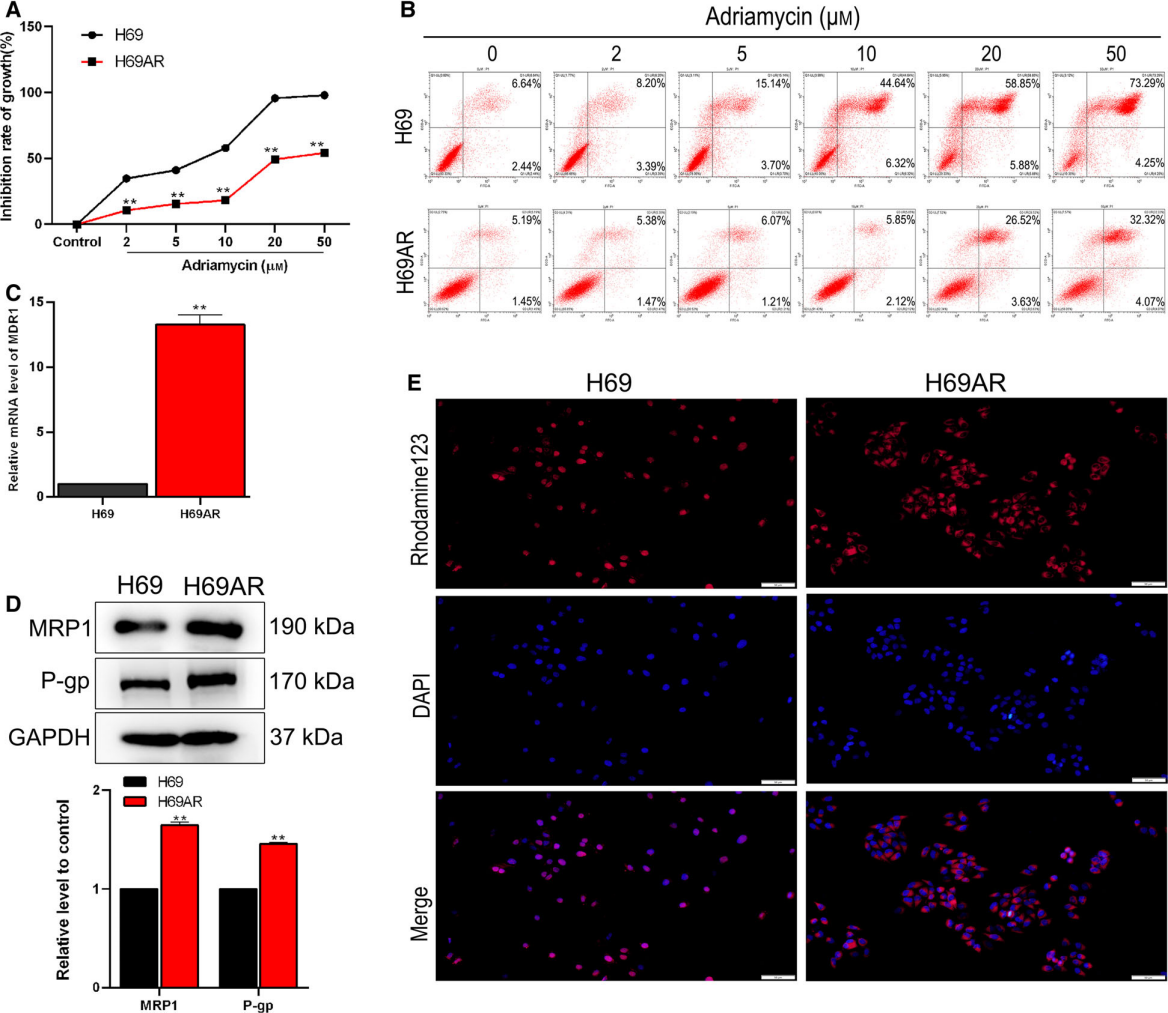
Adriamycin‐resistant profiles of SCLC cells. (A) The growth inhibition rate of H69 and H69AR cells was measured by MTT assay after Adriamycin treatment. **P < 0.01 vs. control. (B) Cell apoptosis was determined by flow cytometry after Adriamycin treatment. (C) Quantitative real‐time PCR analysis of MDR1 expression levels. (D) Western blot analysis of the expression levels of MRP1 and P‐gp; GAPDH was used as a loading control. This part of the results was provided by the contractor. (E) The activity of P‐gp was detected by rhodamine‐123 accumulation assay, sclae bar was 50 μm. Student’s unpaired *t*‐test and one‐way ANOVA were used for statistical analysis. **P < 0.01, vs. H69 group. All results were shown as mean ± SD. Data were obtained from at least three independent experiments.

To assess the drug resistance of SCLC cells treated with Adriamycin, the following experiments were conducted. MTT assay was executed for detecting the growth inhibition effect of Adriamycin (2, 5, 10, 20, and 50 µm) treatment for 48 h in H69 and H69AR cells. The results showed that Adriamycin inhibited the growth of both H69 and H69AR cells in a dose‐dependent manner, and a lower growth inhibition rate was detected in H69AR cells than in H69 cells (*P* < 0.01; Fig. 1A). What is more, to evaluate the Adriamycin resistance of H69AR cells, we calculated and obtained the ratio of the half‐maximal inhibitory concentration of Adriamycin of H69 cells, which was 10 µm. Also, the flow cytometry for the detection of cell apoptosis showed consistent results (Fig. 1B). Besides, the results of quantitative real‐time PCR indicated that the expression level of *MDR1* in H69 cells was lower than H69AR cells (*P* < 0.01; Fig. 1C). The results in Fig. 1D showed that the MRP1 and P‐gp expressions were elevated in H69AR cells (*P* < 0.01). The results of the rhodamine‐123 accumulation assay indicated that P‐gp activity in H69AR cells was higher than H69 cells (Fig. 1E). These findings confirmed that H69AR cells showed superior resistance to Adriamycin in comparison with H69 cells.

### The inflammatory microenvironment promoted drug resistance of SCLC cells by activating the STAT3/VEGF signaling pathway

**Fig. 2:**
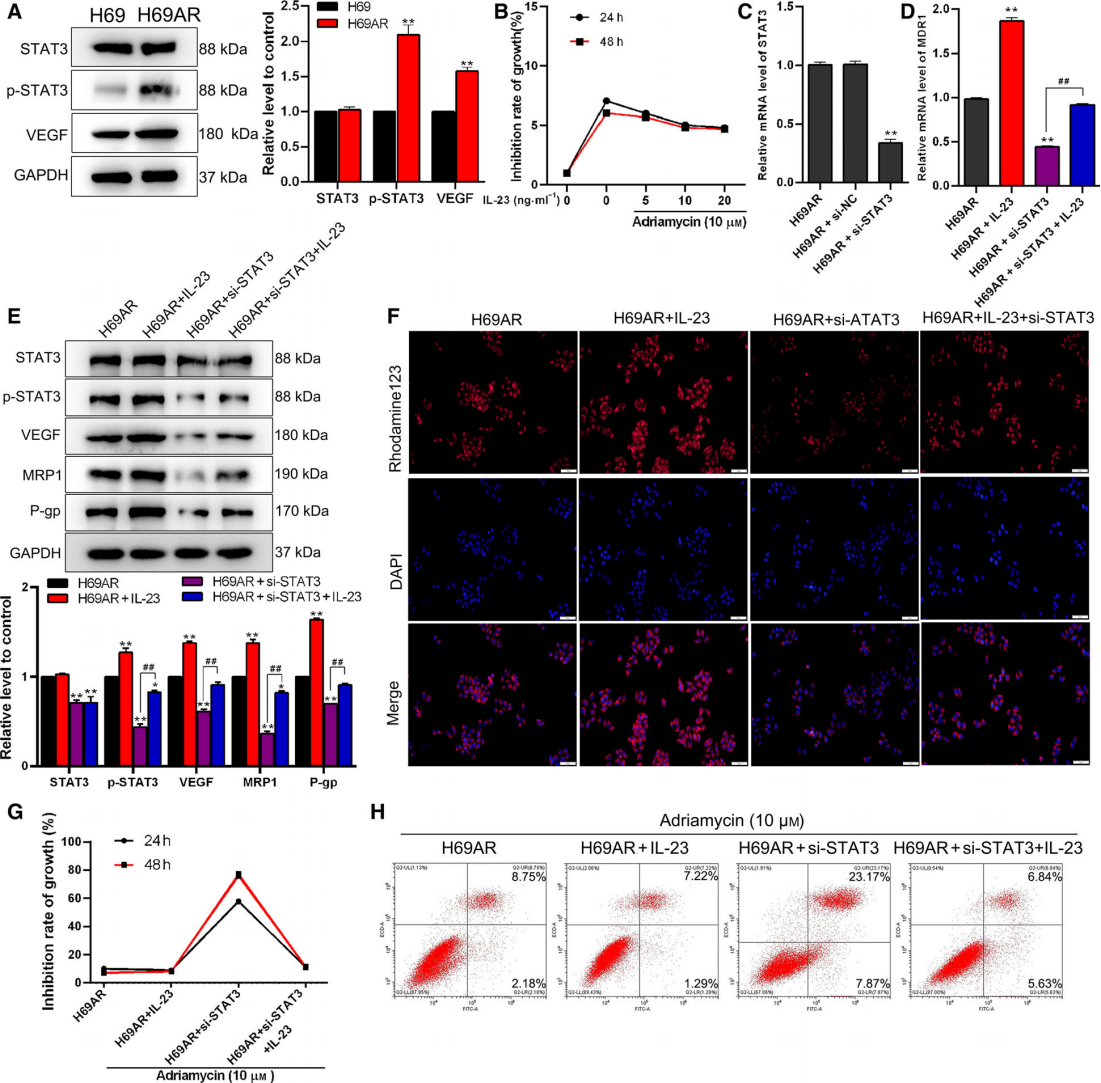
The inflammatory microenvironment plays a role in drug resistance in cancer cells by activating the STAT3/VEGF pathway. (A) Western blot analysis of the expression levels of STAT3, p‐STAT3 and VEGF in H69 and H69AR cells; GAPDH was used as a loading control. This part of the results was provided by the contractor. (B) The cell growth inhibition rate was measured by MTT assay in H69AR cells treated with Adriamycin and IL‐23 for 24 or 48 h. (C) Knockdown of STAT3, the relative mRNA expression level of STAT3 in H69AR cell, was detected by quantitative real‐time PCR. Then cells were divided into four groups (H69AR, H69AR + IL‐23, H69AR + si‐STAT3 and H69AR + IL‐23 + STAT3). (D) Relative mRNA expression levels of MDR1 were detected by quantitative real‐time PCR. (E) The expression levels of STAT3, p‐STAT3, VEGF, MRP1 and P‐gp were detected by western blot. This part of the results was provided by the contractor. (F) The activity of P‐gp was detected by rhodamine‐123 accumulation assay. Scale bar: 50 μm. (G, H) The cell inhibition rate was measured by MTT assay (G), and cell apoptosis was determined by flow cytometry (H). Student’s unpaired *t*‐test and one‐way ANOVA were used for statistical analysis. *P < 0.05, **P < 0.01 vs. H69AR group; ^##^ *P* < 0.01 vs. H69AR + si‐STAT3 group. All results were shown as mean ± SD. Data were obtained from at least three independent experiments.

To investigate the mechanism of the inflammatory microenvironment on the promotion of drug resistance of H69AR cells, we conducted the following experiments. Western blot was used to detect the expression levels of STAT3, p‐STAT3 and VEGF, and the results reported that the levels of p‐STAT3 and VEGF were up‐regulated in H69AR cells compared with H69 cells (*P* < 0.01), which indicated an overactivation of the STAT3/VEGF pathway in drug‐resistant H69AR cells (Fig. 2A). IL‐23 promoted the inflammatory processes in the tumor microenvironment. To detect the effect of the inflammatory microenvironment on drug resistance, we treated the H69AR cells by IL‐23 (0, 5, 10 and 20 ng·mL^−1^) and Adriamycin (10 µm) for 24 and 48 h. The MTT assay was performed to test the growth inhibition rate. As shown in Fig. 2B, the growth inhibition induced by Adriamycin in H69AR cells was significantly relieved under treatment of 10 ng·mL^−1^ IL‐23, and it remained stable under 10–20 ng·mL^−1^ IL‐23 treatments. The results indicated that the inflammatory microenvironment mediated the drug resistance of H69AR, and 10 ng·mL^−1^ IL‐23 was chosen as the appropriate concentration for further analysis. To study the effect of the inflammatory microenvironment on the STAT3/VEGF signaling pathway, the siRNA of *STAT3* was transfected into H69AR to knock down endogenous *STAT3*. The results of quantitative real‐time PCR analysis showed that the *STAT3* expression was dramatically reduced in the H69AR + si‐STAT3 group compared with the H69AR group and H69AR + si‐NC group (*P* < 0.01; Fig. 2C). The results of quantitative real‐time PCR showed that the *MDR1* expression level in the H69AR + IL‐23 group was significantly up‐regulated, whereas that in the H69AR + si‐STAT3 group was down‐regulated. Meanwhile, the decline of the *MDR1* level was reversed by cotreatment with IL‐23 (*P* < 0.01; Fig. 2D). The results of western blot and rhodamine‐123 accumulation assay suggested that the changes of the expression levels of STAT3/VEGF pathway‐related proteins (p‐STAT3, VEGF) and drug resistance‐related proteins (MRP1 and P‐gp), as well as P‐gp activity, were consistent with the earlier trend (Fig. 2E,F). To study the changes of Adriamycin resistance of H69AR cells after treatment by si‐STAT3 and IL‐23, we conducted MTT assay and flow cytometry. After treatment of 10 µm Adriamycin, the growth inhibition rate in the H69AR group was close to that in the H69AR + IL‐23 group, and the growth inhibition rate of H69AR cells was significantly increased by si‐STAT3 transfection, which could be abolished by cotreatment with IL‐23 (Fig. 2G). Similar results were found in cell apoptosis tested by flow cytometry (Fig. 2H). The earlier data showed that the inflammatory microenvironment promoted the drug resistance of H69AR cells through activating the STAT3/VEGF signaling pathway.

### Res played an anti‐inflammatory role to inhibit drug resistance

**Fig. 3:**
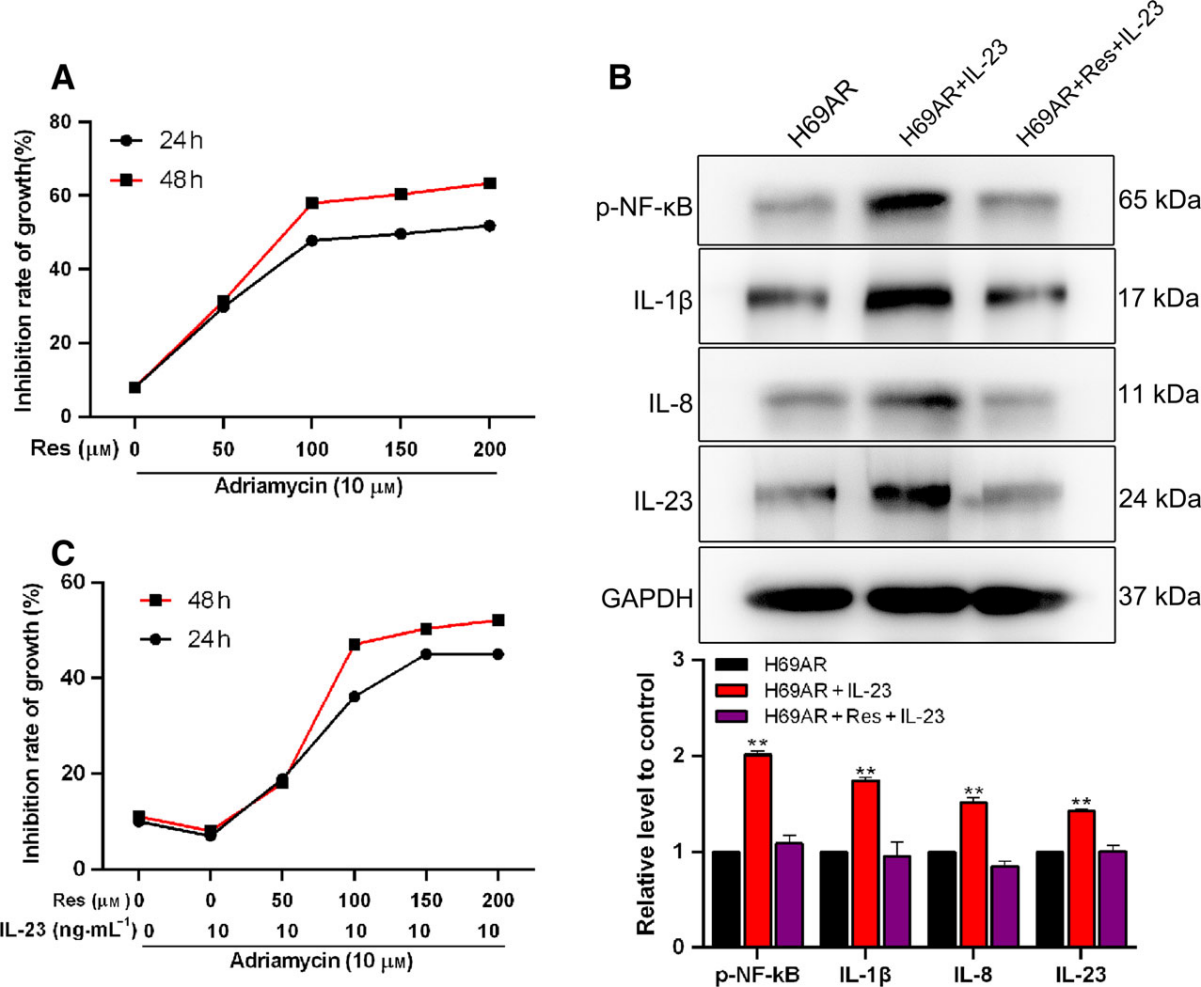
Res plays an anti‐inflammatory role and increases drug sensitivity of H69AR cells. The cell growth inhibition rate was measured by MTT assay in H69AR cells (A) treated with Adriamycin and Res, or (B) treated with Adriamycin, Res and IL‐23 for 24 or 48 h, respectively. (C) Western blot analysis of the expression levels of p‐NF‐κB, IL‐1β, IL‐8 and IL‐23 in H69AR cells; GAPDH was used as a loading control. This part of the results was provided by the contractor. Student’s unpaired *t*‐test and one‐way ANOVA were used for statistical analysis. **P < 0.01 vs. H69AR group. All the results were shown as mean ± SD. Data were obtained from at least three independent experiments.

The H69AR cells were treated by Res (0, 50, 100, 150 and 200 µm) and Adriamycin (10 µm) for 24 and 48 h, respectively; then the Adriamycin resistance of H69AR cells was detected by MTT assay. Fig. 3A shows that the growth inhibition rate of H69AR cells induced by Adriamycin was significantly increased under the treatment of Res (concentration from 0 to 200 µm), and the rate flattened out by Res concentration greater than 100 µm. As shown in Fig. 3B, the H69AR cells were treated by Res (0, 50, 100, 150 and 200 µm), IL‐23 (10 ng·mL^−1^) and Adriamycin (10 µm) for 24 and 48 h, respectively. The results suggested that Res decreased drug resistance of H69AR cells in the inflammatory microenvironments. Besides, 100 µm was selected as the appropriate dose of Res used in the subsequent experiments. To detect the anti‐inflammation role of Res, we assessed the expression levels of inflammation‐related factors. Compared with the H69AR group, the expression levels of p‐NF‐κB, IL‐1β, IL‐8 and IL‐23 were remarkably promoted in the H69AR + IL‐23 group (*P* < 0.01), whereas these increased expression levels were reduced by the addition of Res (Fig. 3C). Therefore, the earlier results suggested that Res played an anti‐inflammatory role and reversed Adriamycin resistance of H69AR cells.

### Res reversed STAT3/VEGF‐mediated drug resistance by ameliorating the inflammatory microenvironment

**Fig. 4:**
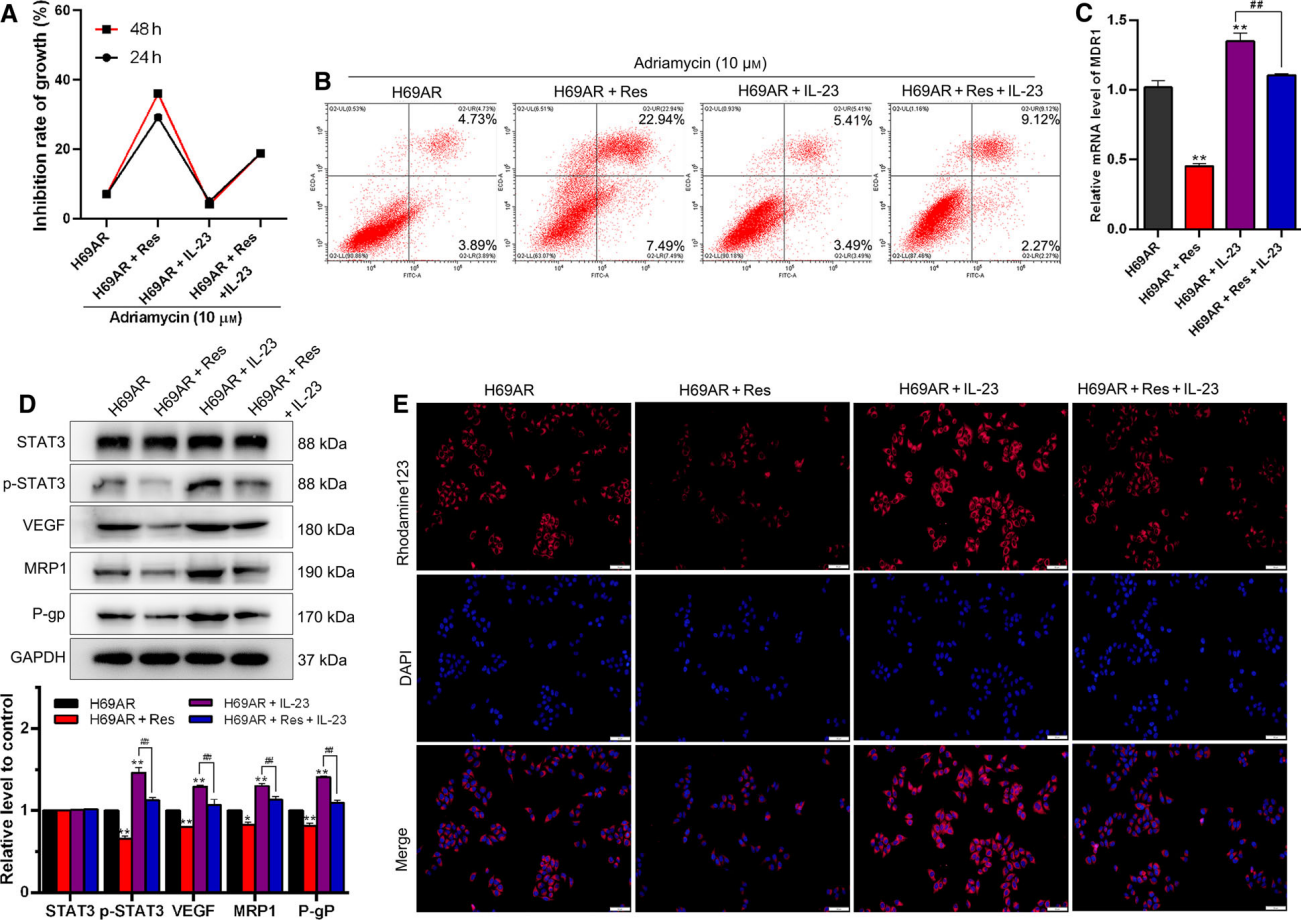
Res reverses STAT3/VEGF‐mediated drug resistance. Cells were divided into four groups (H69AR, H69AR + Res, H69AR + IL‐23 and H69AR + Res + IL‐23). (A) The cell growth inhibition rate was measured by MTT assay in H69AR cells. (B) Cell apoptosis was determined by flow cytometry. (C) The relative mRNA expression level of MDR1 in H69AR cells was detected by quantitative real‐time PCR. (D) The expression levels of STAT3, p‐STAT3, VEGF, MRP1 and P‐gp were detected by western blot. This part of the results was provided by the contractor. (E) The activity of P‐gp was detected by rhodamine‐123 accumulation assay. Scale bar: 50 μm. Student’s unpaired *t*‐test and one‐way ANOVA were used for statistical analysis. *P < 0.05, **P < 0.01 vs. H69AR group; ^##^ *P* < 0.01 vs. H69AR + IL‐23 group. All the results were shown as mean ± SD. Data were obtained from at least three independent experiments.

To investigate whether Res can reverse drug resistance, we performed the following experiments. MTT assay was conducted for detecting the effects of Res (100 µm) on the growth inhibition rate of H69AR cells under Adriamycin (10 µm) treatment. We found that Res reversed Adriamycin resistance in the H69AR + Res group compared with the H69AR group, and the depressed growth inhibition rate under IL‐23 treatment was elevated in the H69AR + IL‐23 + Res group (Fig. 4A). The earlier results suggested that Res reversed the Adriamycin resistance of H69AR cells. The changes in cell apoptosis revealed by flow cytometry were consistent with the earlier results (Fig. 4B). As shown in Fig. 4C, the *MDR1* expression level was significantly decreased in the H69AR + Res group and increased in the H69AR + IL‐23 group, while the elevated *MDR1* expression level in H69AR cells under IL‐23 environment was further declined by Res treatment (*P* < 0.01). The changes in the expression levels of p‐STAT3, VEGF, MRP1 and P‐gp, as well as the P‐gp activity, were consistent with the earlier trend in these groups (*P* < 0.01; Fig. 4D,E). All the findings indicated that Res inhibited the expression levels of inflammatory mediators in the tumor microenvironment and then reduced STAT3/VEGF‐mediated Adriamycin resistance of H69AR cells.

## Discussion

SCLC, as an aggressive disease, is highlighted by high morbidity and mortality [3], and the major obstacle is MDR in SCLC treatment. The H69 cell line is a drug‐sensitive SCLC cell line, while the H69AR cell line was a MDR phenotype. Our results showed that in H69AR cells, the drug‐resistant proteins and transporters were overexpressed compared with H69 cells; after treatment with Adriamycin, H69AR cells developed Adriamycin resistance.

STAT3 targets Bcl‐XL, Cyclin D1, C‐MYC, MCL1 and VEGF, which is involved in various cellular processes [28]. Compared with matched primary tumors, the STAT3 phosphorylation level is increased in drug‐resistant recurrent tumors [29, 30]. Inactivation of STAT3 can overcome drug resistance in lung cancer [31, 32]. In osteosarcoma cells, inhibition of STAT3 increases the sensitivity of chemotherapy‐resistant cells and eliminates drug efflux [33]. Our results also indicated that the drug resistance of lung cancer cells was achieved through activating the STAT3/VEGF signaling pathway, which was consistent with previous research [33]. What is more, the inflammatory microenvironment is involved in the drug resistance of cancer cells. Peng *et al*. [34] have provided evidence that Res regulates drug resistance of SCLC via inhibition of the phosphoinositide 3‐kinase/BMX/STAT3 signaling pathway. The activation of the STAT3 pathway plays a crucial role in the transcriptional regulation of *MDR1* and MRP1 expressions [35]. The activation of STAT3 can increase the expression level of MRP1; therefore, MRP1 overexpression is often detected in various types of cancer. In leukemia cells, the inhibition of the STAT3 pathway can promote the intracellular accumulation of Adriamycin and down‐regulate the expression levels of *MDR1* and MRP1, thus increasing drug sensitivity [36]. Also, the STAT3 signaling pathway is the main intrinsic tumor inflammation pathway, because it is frequently activated in the malignant cells and regulates many tumor inflammatory genes in the tumor microenvironment [37]. Our results indicated that the expression levels of STAT3, P‐gp and MRP1 were reduced in STAT3 knockdown H69AR cells, which was consistent with the earlier results. At the same time, we found that the p‐STAT3, VEGF, P‐gp and MRP1 expressions in the inflammatory microenvironment were elevated, indicating that the inflammatory microenvironment enhanced the drug resistance of SCLC by activating the STAT3/VEGF signaling pathway.

Chronic Res treatment appears to have neuroprotective potential through performing its antioxidative and antiapoptotic functions [38]. The antioxidative and anti‐inflammatory effects of Res were demonstrated in various tumors. In melanoma cells, Res chronic pretreatment was confirmed to inhibit the inflammation, and EMT to induce an antitumor hosting microenvironment for cancer therapies [39]. Furthermore, Res has an antitumor effect in SCLC cells [24]. Bhardwaj *et al*. [26] found that Res inhibited the IL‐6‐induced activation of STAT3. We found that Res played an anti‐inflammatory role by decreasing the expressions of p‐NF‐κB, IL‐1β, IL‐8 and IL‐23, then inactivated the STAT3/VEGF signaling pathway, thus reducing drug resistance of H69AR cells.

In summary, Res overcame STAT3/VEGF‐mediated MDR by suppressing the inflammatory microenvironment, which provided a sound basis for SCLC treatment using Res alone or in combination with other agents in clinical trials. However, the clinical application of Res needs to be further clarified.

## Conflict of interest

The authors declare no conflict of interest.

## Author contributions

CH and LL designed and wrote the paper. ZL and YL analyzed data and performed the experiments. JX conceived the experiments and revised the manuscript. All authors read and approved the manuscript and agree to be accountable for all aspects of the research in ensuring that the accuracy or integrity of any part of the work is appropriately investigated and resolved.

## Data Availability

The datasets used during this study are available from the corresponding author upon reasonable request.
